# Evidence of Multi-Domain Morphological Structures in Living *Escherichia coli*

**DOI:** 10.1038/s41598-017-05897-7

**Published:** 2017-07-18

**Authors:** Sharareh Tavaddod, Hossein Naderi-Manesh

**Affiliations:** Department of Nanobiotechnology, Faculty of Biological Sciences, Tarbiat Modares University, Tehran, P.O. Box 14115-111 Iran

## Abstract

A combination of light-microscopy and image processing was used to elaborate on the fluctuation in the width of the cylindrical part of *Escherichia coli* at sub-pixel-resolution, and under *in vivo* conditions. The mean-squared-width-difference along the axial direction of the cylindrical part of a number of bacteria was measured. The results reveal that the cylindrical part of *Escherichia coli* is composed of multi-domain morphological structures. The length of the domains starts at 150 nm in newborn cells, and linearly increases in length up to 300 nm in aged cells. The fluctuation in the local-cell-widths in each domain is less than the fluctuation of local-cell-widths between different domains. Local cell width correlations along the cell body occur on a length scale of less than 50 nm. This finding could be associated with the flexibility of the cell envelope in the radial versus longitudinal directions.

## Introduction

The mechanism for maintaining the shape of rod-shaped bacteria is a complicated process, and cell shape-maintenance–through the structure of cell envelope–has been an interesting subject for many researchers^1–20^. To date, it is well-known that the cell envelope of the Gram-negative bacteria like *E*. *coli* is comprised of three layers including the outer membrane, inner membrane, and peptidoglycan (PG) layer^8^. Previous studies have revealed that the PG layer, which is a network of peptide and glycan chains, is an essential determinant of the shape of *E*. *coli* ^21–24^. Hence, it is important to elaborate on the architecture of the PG layer, and its flexibility, in parallel, or in advance of the other approaches in studying the cell shape process^22–24^.

Experimentally, studies on the PG layer using optical microscopy have been limited^10, 25^. One of the major problem arises from the lack of proper dyes for tagging the peptide or glycan strands. Fortunately, recent success in designing new tags^26–30^ in combination with developments in optical microscopy techniques, such as fluorescent-photoactivated-localization-microscopy, and stochastic-optical-reconstruction-microscopy should create new opportunities in the coming years.

In this study, we used a combination of optical microscopy (phase-contrast), and image processing^31^ to detect the roughness of cell-envelope in the cylindrical part of 417 bacteria (1.5–3.1 *μ*m), which were grown under steady state conditions^32, 33^. Our measurements were accurate enough to allow us to determine the mean-squared-width-difference (MSWD) along the axial direction of the cylindrical part of bacteria with different lengths. Based on our results, we propose a multi-domain structure in the cylindrical part of *E*. *coli*. Measurements of bacteria with different lengths indicate that the size of these domains linearly depends on the length of the bacteria. Smaller bacteria are composed of domains with smaller lengths rather than fewer domains. In addition, it appears that the fluctuation in the local-cell-widths in each domain is smaller than the fluctuations of local-cell-widths between different domains.

Since it is known that the shape of the cell envelope is modulated mainly by the PG layer, it is tempting to relate our results to the structure of the PG layer. Recently, Nguyen *et al*. studied the growth of the murein sacculus using a coarse-grained model. In the presence of transglycosylases, transpeptidases, and endopeptidases, different structures for the sacculus were predicted by the model^19^. This model suggests that the synthesis of the PG layer could start and progress in different spatial locations, if the cell wall synthesis machinery is suitably coordinated^19^. This modelling study also predicts spatial domains, which are in agreement with our experimental finding of the existence of domains.

## Results

**Figure 1:**
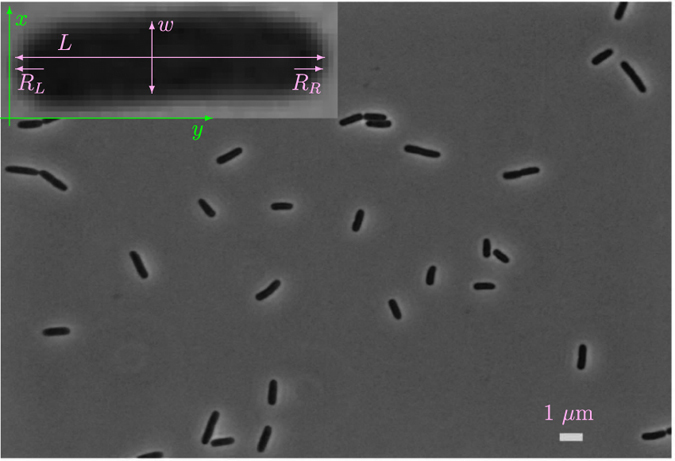
An image of *E*. *coli* cells. A typical image of *E*. *coli* cells with phase-contrast microscopy. *L*, *w*, *R*_*L*_, and *R*_*R*_ indicate the length, width, radius of left and right caps of the single-cell in the inset.

Recent *in vivo* studies of cell shape among different *E*. *coli* strains^34, 35^ have reported spatial heterogeneity in both symmetric and asymmetric aspects of the cell shape, within individual cells. These results are also supported by *in vitro* observation of Vardi *et al*.^36, 37^. To understand this spatial heterogeneity, briefly, the geometry of a typical *E*. *coli* bacterium is approximated by three zones. These are the left cap and right cap (*R*_*L*_ and *R*_*R*_ in the inset of Fig. 1), and the area between two caps (cylindrical-zone). Cells in which radius of the left cap is almost the same as the radius of the right cap, are defined as LR-Symmetric cells.

We studied the fluctuation of the cell-width along the entire cylindrical zone, for individual cells with LR-Symmetry property. To do this, first, a single-cell with LR-Symmetry was selected by measuring the radius of the left and right caps. In order to measure the radius of the left and right caps, poles were detected according to the method of refs ^31, 35^. Briefly, a cell is assumed to be oriented in the *x*–*y* plane such that the width of the bacterium (*w* in the inset of Fig. 1) is along the *x*-direction, and the length of the bacterium (*L* in the inset of Fig. 1) is along the *y*-direction, which is the axial direction of the cell. By considering several cross section cuts along the axial direction of a cell (*y*), and from pole to pole, it is possible to define “local-cell-width” *w*_*j*_, where *j* (=$1,2,3,\ldots ,N$), *N*, and *w*_*j*_ denote the number of cuts relative to the left side pole, total number of cuts, and the local-cell-width at point *j*. Along the axial direction of a cell, the axial distance between two neighboring cross section cuts (or two local-cell-widths) is *δ*_unc_, which is obtained by dividing the length of a cell by the total number of cross section cuts, *N*. The axial distance between any arbitrary pair of cross section cuts (or pair of local-cell-widths) was denoted as the step-length $\ell$, and obtained from $\ell =n{\delta }_{{\rm{unc}}}$, where $n=1,2,3,\ldots ,N-1$ for *N* cross section cuts. Then, the radii of the left (*R*_*L*_) and right (*R*_*R*_) caps were obtained from:

1 $${R}_{L}=\,{\rm{Max}}\,[({w}_{j+\ell }-{w}_{j}) > 2{\delta }_{{\rm{unc}}}],$$

and

2 $${R}_{R}=\,{\rm{Min}}\,[({w}_{j+\ell }-{w}_{j}) < -2{\delta }_{{\rm{unc}}}].$$

**Figure 2:**
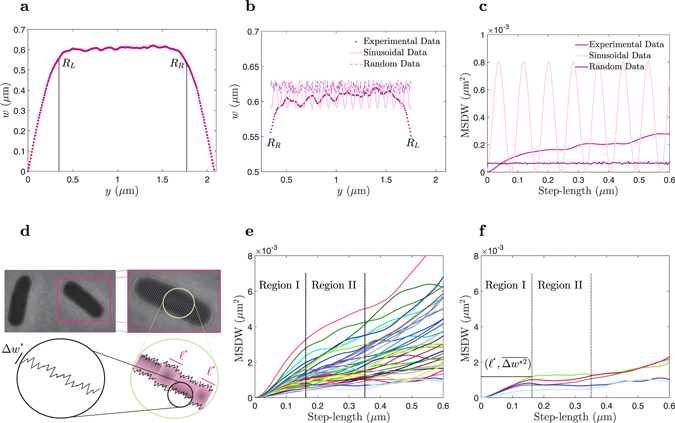
Analysis of the local-cell-width in the cylindrical part of *E*. *coli* cells. (**a**) Variation of the local-cell-width (*w* in Fig. 1) from pole to pole and along the axial direction (*y*) of one cell. (**b**) Variation of the local-cell-width (*w*) of the cylindrical part of a cell: experimental data of the cell, which is shown in (**a**), sinusoidal simulated data, and random simulated data. (**c**) Mean-squared-width-difference (MSWD) of (**b**) (data belongs to one cell). (**d**) Cartoon of multi-domain morphological structures in the cylindrical part of a cell. (**e**) Mean-squared-width-difference *vs*. step-length of 38 cells with 1.5–1.7 *μ*m length. (**f**) Five selected data of (**e**), and the starting point of regime II $({\ell }^{\ast },\overline{{\rm{\Delta }}{w}^{\ast 2}})$.

Taking *N* = 600 (600 local-cell-width), and $\ell =3$ in equation (2), radii of the left and right caps of individual cells (Fig. 2a) were determined and 417 *E*. *coli* cells with LR-Symmetry property were selected^35^. Due to the variation in the length of the selected bacteria (batch culture represents an asynchronous population with cells in all ages), the selected cells were sorted by length and divided into 8 groups. The bacteria within each group had lengths that were identical to within 200 nm (Table 1). Then, the fluctuation of the local-cell-width along the entire length of the “cylindrical part of individual cell” in every group was investigated. As an example, Fig. 2b shows the fluctuation of the local-cell-width in the entire of the cylindrical part of the single-cell of Fig. 2a.

**Table 1:** The number of analyzed cell *N*, in 8 groups.

L (*μ*m)	1.5–1.7	1.7–1.9	1.9–2.1	2.1–2.3	2.3–2.5	2.5–2.7	2.7–2.9	2.9–3.1
*N*	38	108	99	79	53	20	13	7

Division is based on the length of the bacteria *L*.

In order to investigate how the cell width fluctuates, we simulated the local-cell-width (*w*) with 1) a sinusoidal pattern of variation along the cell length, and 2) a random pattern (random noise) of variation along the cell length. As is shown in Fig. 2b, our data for *E*. *coli* more closely matches the simulations with a random variation (random noise) than a known functional pattern.

To elucidate more information about fluctuations in cell width along the length of the cell, the width-difference (WD), squared-width-difference (SWD), and mean-squared-width-difference (MSWD) as a function of step-length $\ell$, along the long axis of a single-cell were studied (See Figs 1 and 2 in Supplementary Information for the probability distribution function of the local-cell-width, and the probability distribution function of difference between local-cell-widths of a single-bacterium in different length range.). By changing the step-length $\ell$, the length scale of correlations in cell width along the cell body could be determined.

More specifically, for a given step-length $\ell$, the squared-width-difference (SWD) was obtained through:

3 $${\rm{\Delta }}{w}_{\ell }^{2}={({w}_{n+\ell }-{w}_{n})}^{2},$$

where ${w}_{n+\ell }$ and *w*_*n*_ are the local-cell-width at points separated by distance $\ell$ (step-length). Then, the MSWD (mean-squared-width-difference) for a given step-length $\ell$, is computed by averaging across all SWD through:

4 $$\overline{{\rm{\Delta }}{w}_{\ell }^{2}}=\overline{{({w}_{n+\ell }-{w}_{n})}^{2}}.$$

Here, ˉ, indicates averaging over all possible SWD for the given $\ell$ in a single-cell.

The MSWD analysis was applied to both our experimental data for a single-cell (Fig. 2a,b) and our simulated (sinusoidal and random) data (Fig. 2b), and as well as for 416 other cells (Fig. 2e,f and Supplementary Figures).

Given that our data for the fluctuations in cell width is most closely fitted by simulations using a random noise model (Gaussian distribution around mean cell-width of the single-cell), we might expect that MSWD *vs*. step-length of each single-cell behaves as a horizontal line. However, interestingly, we observe that, the MSWD *vs*. step-length is in fact a monotonically increasing function. Careful data analysis of 417 bacteria showed that the MSWD *vs*. step-length was not horizontal in the linear scale for any of the bacteria (Figs 2e and 3 in Supplementary Information). Instead, in the regime where the data is statistically accurate (for not too large step-lengths), two meaningful regimes I and II were seen (Figs 2e and 3 in Supplementary Information). Regime I, did not depending on which single-cell was chosen to be analyzed. However, regime II could take the form of a plateau function or a monotonically increasing function, depending on which single-cell was chosen to be analyzed.

**Figure 3:**
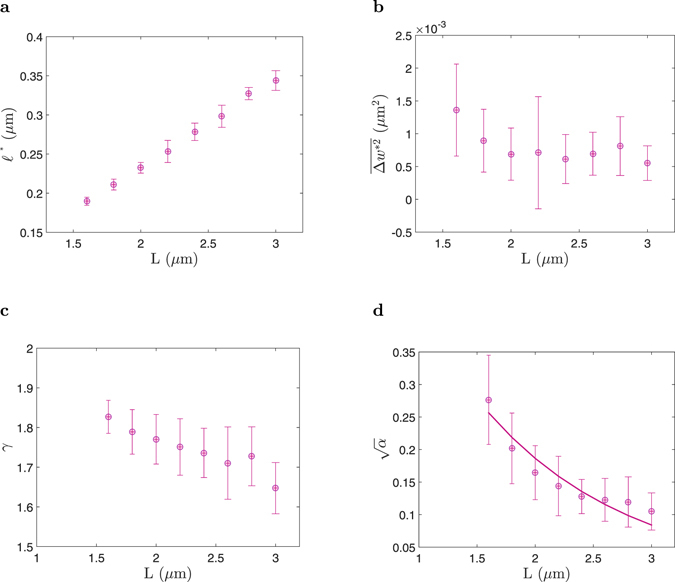
Characterization of multi-domain morphological structures based on the length of *E*. *coli* cells. Data of panels are from 417 cells (Table 1) with different lengths *L*, and are expressed as mean ± standard error of mean. (**a**) Length of the domains ${\ell }^{\ast }$ (starting point of the regime II), based on the length of cells. (**b**) MSWD of the local-cell-width in a domain $\overline{{\rm{\Delta }}{w}^{\ast 2}}$ (starting point of the regime II), based on the length of cells. (**c**) Exponent of the fitted polynomial function *γ* (${\rm{MSWD}}=\alpha {\ell }^{\gamma }$), to the first 20 points based on the length of cells. (**d**) Coupling constant $\sqrt{\alpha }$ (${\rm{MSWD}}=\alpha {\ell }^{\gamma }$), based on the length of cells. The error bars indicate the standard error of mean of each bin.

The starting point of the regime II (Fig. 2f), is denoted by $({\ell }^{\ast },\overline{{\rm{\Delta }}{w}^{\ast 2}})$. The crossover length ${\ell }^{\ast }$ between regimes I and II was obtained for “all” single-cells in “each” group. To do that, the MSWD *vs*. step-length data both in the log-log scale (Fig. 4 in Supplementary Information), and the linear scale were used to find the “local” maximum of $\overline{{\rm{\Delta }}{w}_{\ell }^{2}}$. It was done by comparing the absolute value of each $\overline{{\rm{\Delta }}{w}_{\ell }^{2}}$ with $\overline{{\rm{\Delta }}{w}_{\ell +10}^{2}}$. The $\ell$ corespondent to the “local” maximum of MSWD *vs*. step-length, was defined as ${\ell }^{\ast }$ and considered as a starting point of the regime II for each single-cell. ${\ell }^{\ast }$ for all single-cells in each group were obtained, and the mean value of ${\ell }^{\ast }$ (±standard error of mean) for each group is shown in Fig. 3. Clearly, the mean value of ${\ell }^{\ast }$ in each group, linearly depends on the length of the cells in the group. To understand more about the meaning of ${\ell }^{\ast }$, the behavior of its correspondent $\overline{{\rm{\Delta }}{w}^{\ast 2}}$, was studied.

At the level of resolution that we can obtain by combining optical microscopy and image processing, and on length scales shorter than ${\ell }^{\ast }$ in MSWD *vs*. step-length of each single-cell (Figs 2e and 3 in Supplementary Information), the relation of $\overline{{\rm{\Delta }}{w}^{2}} < \overline{{\rm{\Delta }}{w}^{\ast 2}}$ is seen. This indicates that on length scales shorter than ${\ell }^{\ast }$, cell width was relatively constant along the cell length. We suppose that the cylindrical part of the bacterium is made up of several domains, and within each domain the cell width is approximately constant (Fig. 2d). It is not possible to quantify the number of these domains in each single-cell but, by considering the length of the bacteria, and knowing the typical mean value of ${\ell }^{\ast }$ of each group, the estimated number of domains must be less than 10. Figure 3a shows, as a bacterium grows (length increases), the number of domains does not increase but rather, the length of the domains (the mean value of ${\ell }^{\ast }$) increases. For step-lengths smaller than ${\ell }^{\ast }$ ($\ell  < {\ell }^{\ast }$), the MSWD was mainly composed of a sum of the SWDs (${w}_{n+\ell }$ and *w*_*n*_), corresponding to the same domain. In contrast, for step lengths larger than ${\ell }^{\ast }$ ($\ell  > {\ell }^{\ast }$), the MSWD was mainly consists of a sum of ${w}_{n+\ell }$ and *w*_*n*_, corresponding to different domains. We suppose that for a bacterium whose MSWD *vs*. step-length has a longer plateau ($\ell  > {\ell }^{\ast }$), the widths of distant domains are almost the same, and the cylindrical part of the bacterium more or less, has a constant width on a longer length-scale. In the next step, $\overline{{\rm{\Delta }}{w}^{\ast 2}}$ of bacteria were obtained, and as Fig. 3b shows, these values were about $\overline{{\rm{\Delta }}{w}^{\ast 2}}\sim 8\times {10}^{-4}\,\mu {{\rm{m}}}^{2}$, in each group. Thus, the mean fluctuation (absolute) of the local-cell-width along the axial direction of a cell is on the scale of $\overline{{\rm{\Delta }}w}\sim 30\,{\rm{nm}}$, independent of the cell length (Fig. 3b). Consistent with this, our simulations shown in Fig. 2b, assumed *w* = 600 nm, and $\overline{{\rm{\Delta }}w}=20\,{\rm{nm}}$, in the simulation of (1) a sinusoidal fluctuation pattern, and (2) a random fluctuation pattern of cell-width.

Regime I was elucidated by fitting the polynomial function ${\rm{MSWD}}=\alpha {\ell }^{\gamma }$, to the first 20 points of our MSWD *vs*. step-length data. The coefficient *α*, and the exponent *γ*, was extracted for every bacterium, and the mean value of $\sqrt{\alpha }$, and *γ* of each group, as a function of cell-length are shown in Fig. 3c,d. The exponent of the polynomial function is consistent across our data, and indicates the universal behavior of $\overline{{\rm{\Delta }}{w}^{2}}\sim {\ell }^{2}$. It would seem that on the length-scale of $\tilde{\ell }$, where $0 < \tilde{\ell } < 50\,\,{\rm{nm}}\ll {\ell }^{\ast }$, there is a local correlation in cell width, where $|{\rm{\Delta }}w|\sim \tilde{\ell }$. Our analysis was based on the image of the largest longitudinal-cut cross of individual cells (2D image of the cell envelope of individual cells). Thus, because of the symmetry in the axial direction of *E*. *coli*’s shape, we imaged the largest longitudinal-cut with an arbitrary azimuthal angle (in the cylindrical coordinate system) of every cell. Hence, it might be possible to conclude that in every localized bulk of cell envelope with dimensions $w\times w\times \tilde{\ell }$, the fluctuation in cell-width (Δ*w*) of a pair of points with axil distance of $\tilde{\ell }$, linearly relates to the axial distance of those points. This linear relation between Δ*w* and $\tilde{\ell }$, could be evidence of coupling behavior at a length scale much smaller than 50 nm ($\sqrt{\alpha }$ as a coupling constant).

An important point that should be mentioned here is that the localized-cell-width was measured with 1–5 nm spatial resolution. This high level of spatial resolution was made possible by combining image processing and digital optical imaging. However, the resolution of our measurements of $\overline{{\rm{\Delta }}w}$ (and following that in $\overline{{\rm{\Delta }}{w}^{2}}$) was less good (larger than 1–5 nm), but still much smaller than 50 nm, where 34 nm (see Sec. Methods) is the spatial resolution from the digital optical imaging (the mapped size of a pixel in image processing) in *x* and *y* directions. Hence, the concept of coupling is still valid but, its linear relation and the strongness of the coupling could vary, and it would be interesting further investigation.

Finally, as Fig. 3d shows, the function $\sqrt{\alpha }=b\,\exp \,(\tfrac{L}{\lambda })$ was fitted to the coupling constant, when the length of bacteria varied in the range of 1.5 ≤ *L* ≤ 3.1 *μ*m, and *λ* ~ 1.25 *μ*m is the length that the coupling constant decreases to its half value. This shows that, as a new born bacterium grows, the domains, which are initially small in size, grow in length such that the radius of the new added sacculus material is correlated to the older sacculus material (at longitudinal distance much smaller than 50 nm) with a coupling constant $\sqrt{\alpha }$. The coupling constant decreases exponentially, and *λ* could be considered as a “decoupling-length”. Our finding could be a platform to investigate why sacculi of longer bacteria show more curvature in their shape^10^.

## Discussion

We studied fluctuations in the cell width along the axial direction of 417 individual *E*. *coli* cells. Our analysis of the mean-squared-width-difference has revealed that the cylinderical part of an *E*. *coli* bacterium could be made of several domains. The length of the predicated domains starts at 150 nm, and it linearly increases in length up to 300 nm. Newborn cells showed smaller domains rather than fewer domains, and as cells grew, the length of the domains increased.

In addition, at a length scale smaller than 50 nm along the cell envelope, the size of the cell-width is coupled to the sizes of the neighboring cell-widths, which could e associated with the flexibility in the radial direction of the cell envelope relative to the longitudinal direction of the cell envelope.

These findings could be of considerable importance with regard to the growth of domains during the dynamics of cell-growth. Furthermore, since the shape of bacteria is strongly modulated by the architecture of the PG layer, it might be possible to interpret our finding as a fine structure of the PG layer, which needs more investigation in future.

The advantage of the present report is, while the experiment was done under *in vivo* condition, the results had been obtained without applying external forces, torques, and pressures.

## Methods

The mutated *E*. *coli* strain HCB137 (F *thi*-*1 his*-*4 met*F(Am)159 *eda50rpsL 136srl*::Tn*10* Δ(*flhC*-*flhA*)), was obtained kindly from K. Fahrner, Department of Molecular and Cellular Biology, Harvard University, Cambridge, MA. The strain is not motile because of lacking flagella (Δ(*flhC*-*flhA*)) and the MotA-MotB complex (*motA* and *motB*). Cells were grown at 21 in TY medium (10 g tryptone, 5 g yeast extract, and 5 g of NaCl per liter, and *p*H = 7)^35, 38–42^. The cells were kept in the exponential-growth-regime for more than 20 generations by diluting periodically in the same pre-warmed medium. The maximum optical density (Nano drop 2000c Spectrophotometer, Thermo Scientific) of a cell culture before each dilution was 0.5 at 450 nm, which is more sensitive wavelength to detect low cell number. The mentioned growth method achieved a “homogenous bacterial culture”. The images of the present study were recorded from the cell culture flasks, which had been grown periodically, and diluted in 1.2 days after 20 generations.

At optical densities 0.1 (at 450 nm), sampling and chambers prepration from a certain flask had started and it continued about 150 min. Then, the optical density of the cell culture during imaging was in the range of 0.1–0.3 (at 450 nm). During this time interval, a drop of 3.5 − 4 ± 0.5 *μ*l bacterial culture were pipetted onto a clean microscope slide. Immobilization to the glass surface was done by a clean coverslip (with 22 mm × 22 mm surface area). The coverslip was gently placed on top of the drop. However, for unknown reasons in some cases, cells were not immobilized by attachment and those samples, which were checked by microscope, were not used for imaging. To prevent shrinkage or any additional possible change in the morphology of cells, VALAP (equal weight of Vaseline, Lanolin, and Paraffin) was used to seal the chamber. Hence, during performing digital-camera-microscopy, cells were alive, and in principle, could continue to grow. To avoid imaging of cells, which were in the stationary-phase (due to the lack of oxygen), each prepared sample was used only 20 min. Since the doubling time of the cells (*T*_*D*_ = 110 ± 17 min) was a lot larger than the time of imaging (20 min), during imaging time cells did not run out of oxygen. To remedy the complicatedness of the effect of cell division in our results, the cells were chosen (had been judged by eye) from those cells whose shapes had not shown sign of cell constriction (pre-divisional state of the growth).

Imaging was done by an inverted phase-contrast microscope (IX81, Olympus) with a 100×, 1.4 NA phase oil objective in combination of a digital-camera (DP72, Olympus). The images (1360 × 1024 pixels) were taken from different parts of the chamber, and far from the edges. To the best of our observation, the lengths of the immobilized cells were always in the focal plane, which leads us to assume that the projected area of a cell in an image is the largest longitudinal-cut cross the cell.

The method of image processing was the same as the method of Guberman *et al*.^31, 35^. The edge position was defined based on the defining a binary threshold value. Then, the method of interpolation of contours (the roughness problem due to the pixilated border), was applied, which is based on choosing a specific threshold in the manner that causes less sensitivity to find the total area of a bacterium. All image processing and data analysis was done using MATLAB (The MathWorks, Natick, MA). Positions of poles, and cell-border were extracted from images with the final spatial resolution of 34 nm, in *x* and *y* directions. The interpolated contour contained a series of discrete points with 1–5 nm spatial resolution.

## Electronic supplementary material

Supplementary Information
