# Supplementary material for: Evidence of Multi-Domain Morphological Structures in Living Escherichia coli
Source: Sci Rep. 2017 Jul 18;7:5660. doi: 10.1038/s41598-017-05897-7 (PMC5516040; doi:10.1038/s41598-017-05897-7)
Supplement: Supplementary file 1 — Supplementary Information [file 41598_2017_5897_MOESM1_ESM.pdf]

Evidence of Multi-Domain Morphological Structures in Living *Escherichia coli*

Sharareh Tavaddod[1] and Hossein Naderi-Manesh[1*]
[1]Department of Nanobiotechnology, Faculty of Biological Sciences, Tarbiat Modares University, Tehran, P.O. Box 14115-111, Iran. *naderman@modares.ac.ir

Supplementary Figures:

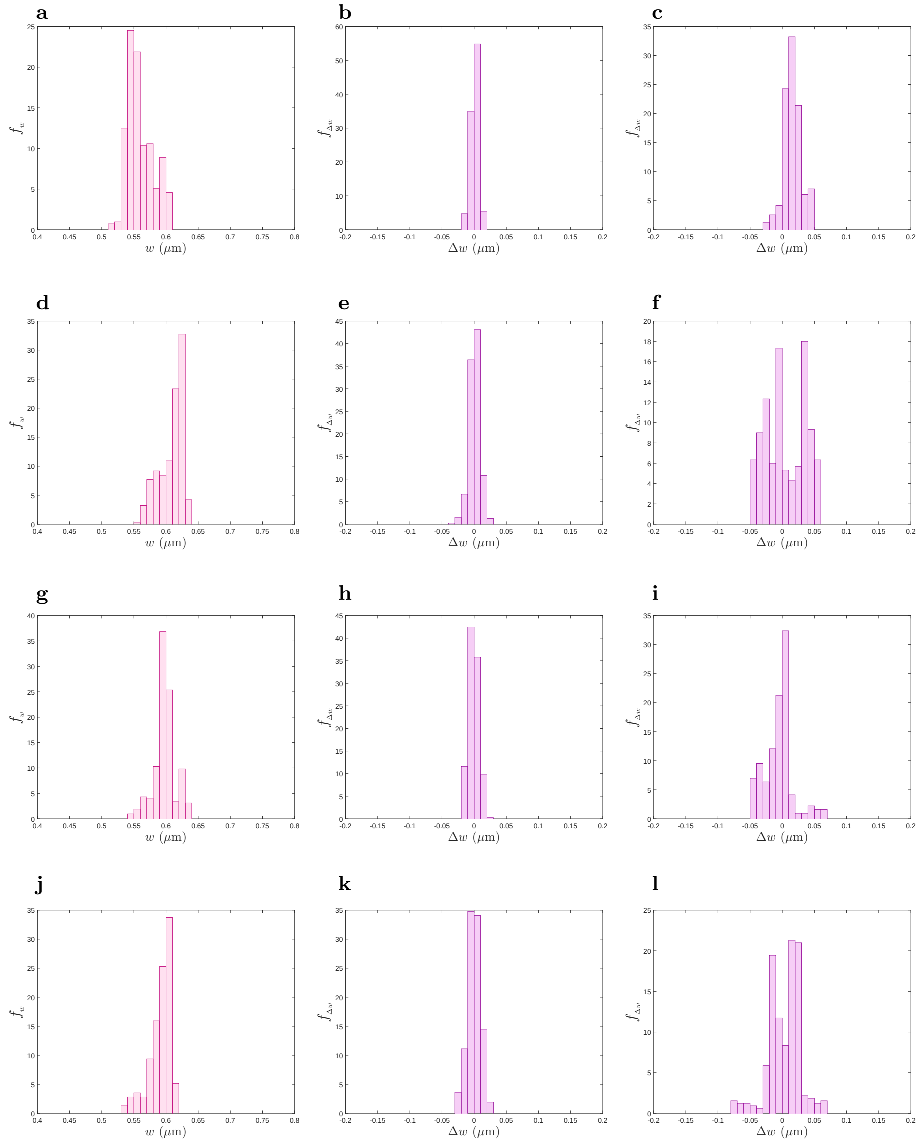

Figure 1. Probability distribution function of the local-cell-width of single bacteria in different length range. Curves of each row are belonged to the statistical analysis of local-cell-width (N = 600) of a single

bacterium with length range of (a-c) $L = 1.5$-1.7 μm,(d-f) $L = 1.7$-1.9 μm, (g-i) $L = 1.9$-2.1 μm, (j-l) $L = 2.1$-2.3 μm. First image from left in each row (first column from left) presents the probability distribution function of the local-cell-width (f_w) in the cylindrical region of a single-cell. Second image from left in each row (second column from left) presents the probability distribution function of difference between local-cell-widths ($f_{\Delta w}$) with step-length $\ell = 10$. Third image from left in each row (third column from left) presents the probability distribution function of difference between local-cell-widths ($f_{\Delta w}$) with step-length $\ell = 100$.

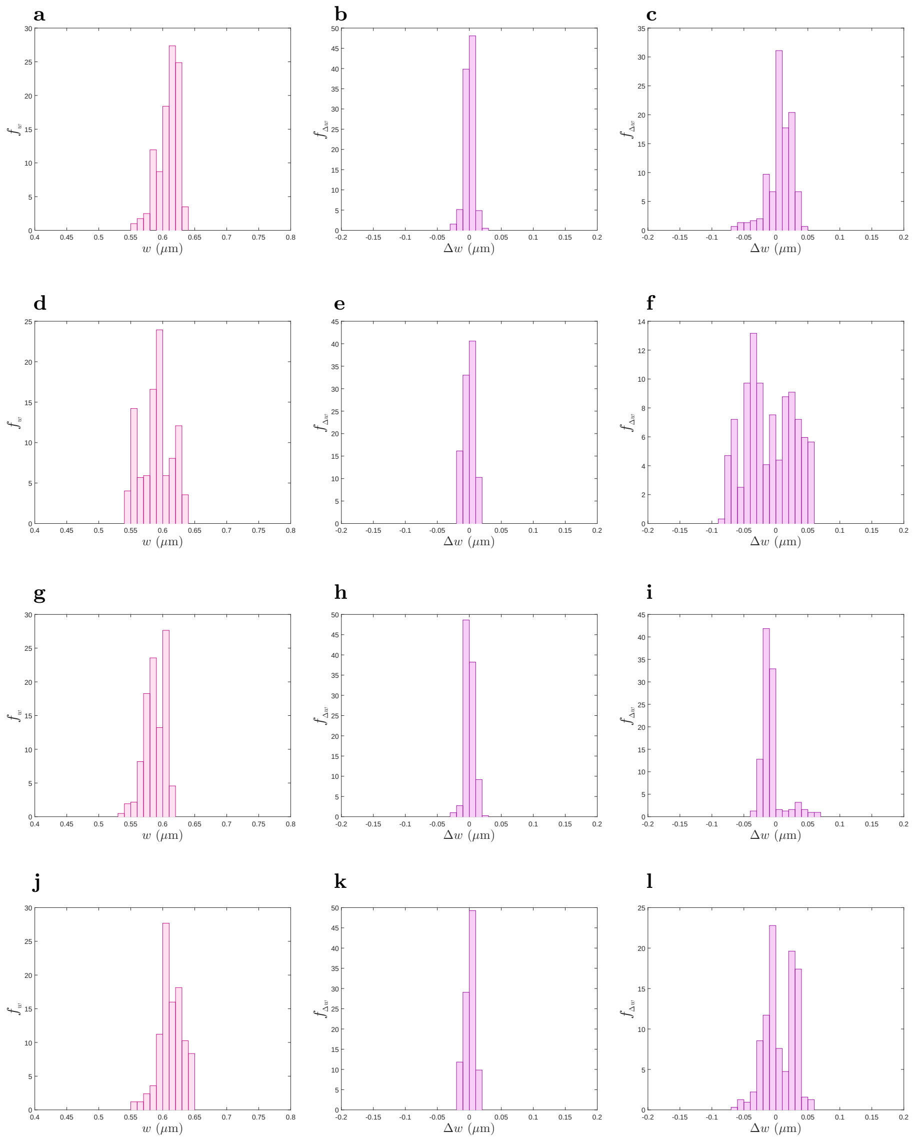

Figure 2. Probability distribution function of the local-cell-width of single bacteria in different length range. Curves of each row are belonged to the statistical analysis of local-cell-width (N = 600) of a single bacterium with length range of (a-c) L = 2.3-2.5 μm,(d-f) L = 2.5-2.7 μm, (g-i) L = 2.7-2.9 μm, (j-l) L = 2.9-3.1 μm. First image from left in each row (first column from left) presents the probability distribution function of the local-cell-width (f_w) in the cylindrical region of a single-cell. Second image from left in each row (second column from left) presents the probability distribution function of difference between local-cell-widths ($f_{\Delta w}$) with step-length ℓ = 10. Third image from left in each row (third column from left) presents the probability distribution function of difference between local-cell-widths ($f_{\Delta w}$) with step-length ℓ = 100.

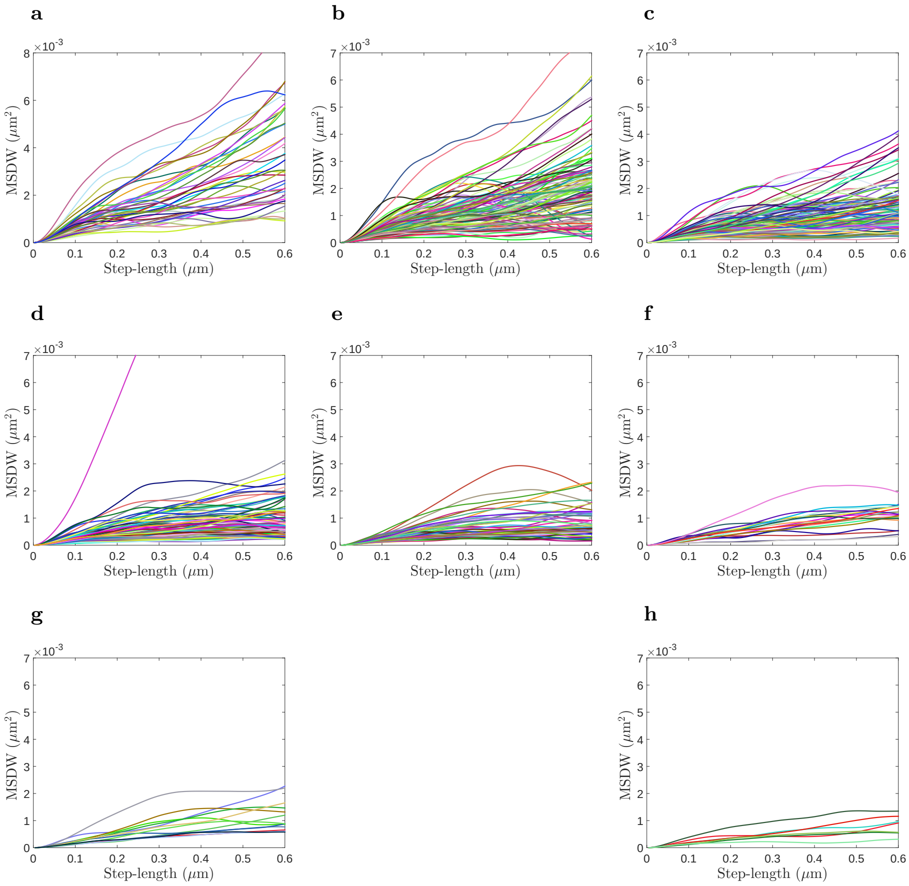

Figure 3. Mean-squared-width-difference *vs.* step-length of cells with different length. Mean-squared-width-difference *vs.* step-length of cells with length range (a) L = 1.5-1.7 μm, (b) L = 1.7-1.9 μm, (c) L = 1.9-2.1 μm, (d) L = 2.1-2.3 μm, (e) L = 2.3-2.5 μm, (f) L = 2.5-2.7 μm, (g) L = 2.7-2.9 μm, (h) L = 2.9-3.1 μm.

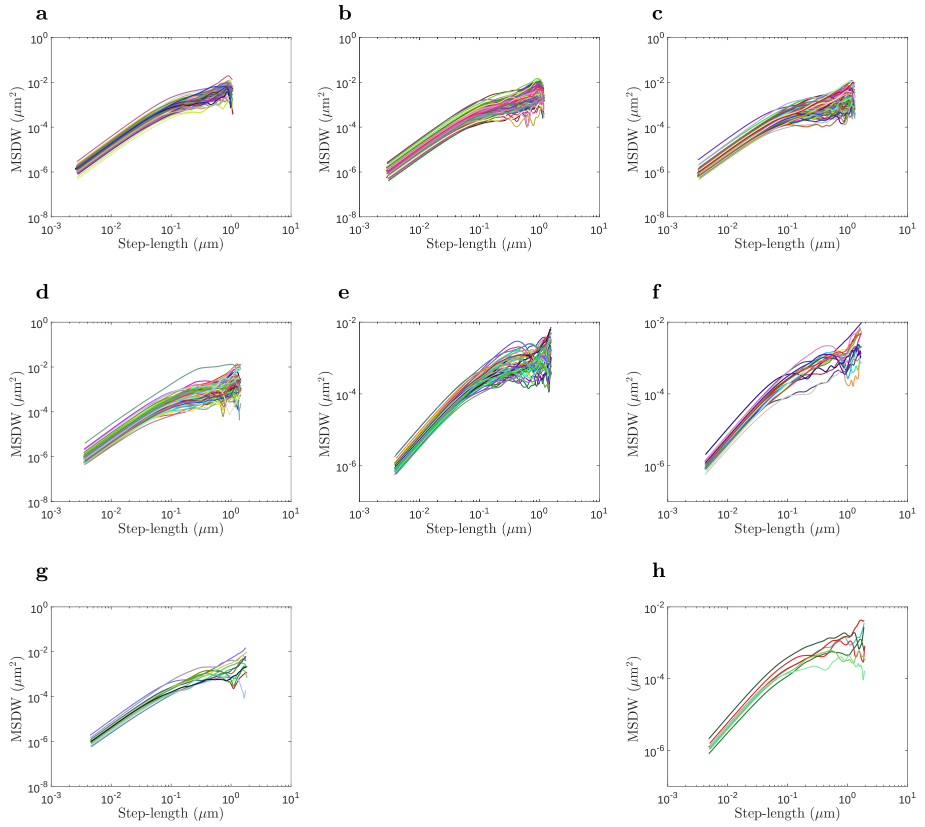

Figure 4. Mean-squared-width-difference *vs.* step-length of cells with different length in logarithmic scale.
Mean-squared-width-difference *vs.* step-length in logarithmic scale of cells with length range (a) L = 1.5-1.7 μm, (b) L = 1.7-1.9 μm, (c) L = 1.9-2.1 μm, (d) L = 2.1-2.3 μm, (e) L = 2.3-2.5 μm, (f) L = 2.5-2.7 μm, (g) L = 2.7-2.9 μm, (h) L = 2.9-3.1 μm.
